# Cell growth and lipid accumulation of a microalgal mutant *Scenedesmus* sp. Z-4 by combining light/dark cycle with temperature variation

**DOI:** 10.1186/s13068-017-0948-0

**Published:** 2017-11-09

**Authors:** Chao Ma, Yan-Bo Zhang, Shih-Hsin Ho, De-Feng Xing, Nan-Qi Ren, Bing-Feng Liu

**Affiliations:** State Key Laboratory of Urban Water Resource and Environment, Harbin Institute of Technology, P.O. Box 2614, 73 Huanghe Road, Harbin, 150090 China

**Keywords:** Light/dark cycle, Lipid production, Autotrophic, Mixotrophic, *Scenedesmus* sp. Z-4

## Abstract

**Background:**

The light/dark cycle is one of the most important factors affecting the microalgal growth and lipid accumulation. Biomass concentration and lipid productivity could be enhanced by optimization of light/dark cycles, and this is considered an effective control strategy for microalgal cultivation. Currently, most research on effects of light/dark cycles on algae is carried out under autotrophic conditions and little information is about the effects under mixotrophic cultivation. At the same time, many studies related to mixotrophic cultivation of microalgal strains, even at large scale, have been performed to obtain satisfactory biomass and lipid production. Therefore, it is necessary to investigate cellular metabolism under autotrophic and mixotrophic conditions at different light/dark cycles. Even though microalgal lipid production under optimal environmental factors has been reported by some researchers, the light/dark cycle and temperature are regarded as separate parameters in their studies. In practical cases, light/dark cycling and temperature variation during the day occur simultaneously. Therefore, studies about the combined effects of light/dark cycles and temperature variation on microalgal lipid production are of practical value, potentially providing significant guidelines for large-scale microalgal cultivation under natural conditions.

**Results:**

In this work, cell growth and lipid accumulation of an oleaginous microalgal mutant, *Scenedesmus* sp. Z-4, were investigated at five light/dark cycles (0 h/24 h, 8 h/16 h, 12 h/12 h, 16 h/8 h, and 24 h/0 h) in batch culture. The results showed that the optimal light/dark cycle was 12 h/12 h, when maximum lipid productivity rates of 56.8 and 182.6 mg L^−1^ day^−1^ were obtained under autotrophic and mixotrophic cultivation, respectively. Poor microalgal growth and lipid accumulation appeared in the light/dark cycles of 0 h/24 h and 24 h/0 h under autotrophic condition. Prolonging the light duration was unfavorable to the production of chlorophyll a and b, which was mainly due to photooxidation effect. Polysaccharide was converted into lipid and protein when the light irradiation time increased from 0 to 12 h; however, further increasing irradiation time had a negative effect on lipid accumulation. Due to the dependence of autotrophically cultured cells on light energy, the light/dark cycle has a more remarkable influence on cellular metabolism under autotrophic conditions. Furthermore, the combined effects of temperature variation and light/dark cycle of 12 h/12 h on cell growth and lipid accumulation of microalgal mutant Z-4 were investigated under mixotrophic cultivation, and the results showed that biomass was mainly produced at higher temperatures during the day, and a portion of biomass was converted into lipid under dark condition.

**Conclusions:**

The extension of irradiation time was beneficial to biomass accumulation, but not in favor of lipid production. Even though effects of light/dark cycles on autotrophic and mixotrophic cells were not exactly the same, the optimal lipid productivities of *Scenedesmus* sp. Z-4 under both cultivation conditions were achieved at the light/dark of 12 h/12 h. This may be attributed to its long-term acclimation in natural environment. By combining temperature variation with optimal light/dark cycle of 12 h/12 h, this study will be of great significance for practical microalgae-biodiesel production in the outdoor conditions.

**Electronic supplementary material:**

The online version of this article (10.1186/s13068-017-0948-0) contains supplementary material, which is available to authorized users.

## Background

The increasing demand for alternative energy sources attracts the interest in biofuel production. Microalgal biodiesel is considered a third-generation biofuel, which shows great potential for sustainable production, and it could reduce the competition with food crops [1]. Microalgal growth does not require arable land; however, the cost of biodiesel production is still expensive, which is mainly due to the algal cultivation and harvesting process. To solve this issue, a series of strategies have been applied, such as screening of lipid-rich microalgal strains [2], lipid and biomass accumulation coupled with wastewater [3, 4], and optimization of environmental factors [5–7]. Among these strategies, options of environmental factors (pH, light intensity and duration, temperature, etc.) are of great importance to overall biomass and lipid production by microalgal cells. On one hand, they could affect the cell growth and lipid synthesis of microalgal strains with high lipid content. On the other hand, they play an important role in the potential of microalgal cultivation using various wastewaters. Growth of different algal species has been enhanced by regulation of light irradiation and temperature in many studies. For example, the maximum biomass of 0.992 g L^−1^ and CO_2_ fixation rate of 0.326 g L^−1^ day^−1^ were obtained under optimal light intensity of 13,000 lx for *Scenedesmus obliquus* [8], and the best temperature and irradiance for cell growth of *Selenastrum minutum* were 35 °C and 420 μmol m^−2^ s^−1^, respectively [9]. Therefore, determination of optimal light and temperature conditions is deemed an effective control strategy for microalgal cultivation.

Light, which is the energy source of photosynthesis, is a key factor for microalgal growth and carbon dioxide fixation [10]. The quality, intensity, and period of light all have significant effects on the amount of light energy received by microalgae, and these effects are species-dependent. A *Nannochloropsis* sp. strain exhibited maximum cell concentration and lipid content under light intensity of 100 μmol m^−2^ s^−1^ and light/dark cycle of 18 h/6 h [11]; however, optimal growth rate and biomass productivity of *Neochloris terrestris* were obtained at the light/dark cycle of 12 h/12 h with light intensity of 60 μmol m^−2^ s^−1^ [12]. It was also reported that pathways including carbon fixation, glucan biosynthesis, and lipid biosynthesis were up-regulated during the light period, and TCA cycle and lipid β-oxidation occurred during the dark period, when *Phaeodactylum tricornutum* was acclimated to a light/dark cycle of 16 h/8 h [13]. The results demonstrated that the light/dark cycle indeed played an important role in cellular metabolism of microalgae.

Cellular metabolism related to microalgal growth and lipid production differs between autotrophic and heterotrophic conditions. Under autotrophic cultivation, light is the most important energy source for microalgal photosynthesis, and biomass production, lipid accumulation, and CO_2_ fixation strongly depend on the supply of light energy. In contrast, under heterotrophic cultivation, CO_2_ is replaced by organic matter as carbon sources, no light energy is needed, and better performance of biomass production and lipid synthesis has been gained when microalgal cells are fed with glucose [14], glycerol [15], and sucrose [16]. When microalgal growth is provided with both organic carbon source and light energy, this mode is often called mixotrophic cultivation. The accumulation of biomass, lipid, or chlorophyll under mixotrophic conditions is higher than those under autotrophic and heterotrophic conditions, which has led to increasing interest in research on mixotrophic cultivation of microalgal strains [17–19]. Due to presence of organic carbon sources, the demand of mixotrophic cells for light is not as strong as that of autotrophic cells. A study has reported that photosynthesis of *Chlorella sorokiniana* was regulated differently under mixotrophic conditions compared to that under autotrophic conditions [20], implying different pathways in mixotrophically and autotrophically cultured cells. However, scarce information on the correlation between light/dark cycles and biomass and lipid production under mixotrophic cultivation could be found in the literature. Therefore, it is crucial to simultaneously investigate the effects of light/dark cycles on cellular metabolism of microalgal strains under different cultivation modes.

Temperature is also an important parameter for microalgal growth, and the optimal temperature range of 20–30 °C is observed for different algal species [21]. Enhanced biomass concentration and lipid productivity have been obtained by optimization of temperature [22, 23]. However, to date no studies about lipid accumulation and cell growth under conditions of temperature variation have been reported, which agrees well with actual lipid synthesis under natural conditions. Therefore, studies on lipid production under continuous temperature variation urgently need to be investigated and this could be beneficial to research on lipid synthesis in outdoor conditions.

In this work, a previously screened oleaginous microalgal mutant *Scenedesmus* sp. Z-4 was used [24] and desirable biomass and lipid productivity were observed under heterotrophic conditions. This study aims to explore the effects of different light/dark cycles on cell growth and lipid accumulation of *Scenedesmus* sp. Z-4 under autotrophic and mixotrophic conditions. The photosynthetic efficiency and variation of cellular components were discussed to characterize cellular metabolism at different light/dark cycles under these two cultivation modes. More importantly, the combined effects of temperature variation with optimal light/dark cycle of 12 h/12 h on cell growth and lipid accumulation of mixotrophically cultured mutant Z-4 were also studied.

## Results and discussion

### Effects of light/dark cycles on cell growth and lipid accumulation of microalgal mutant *Scenedesmus* sp. Z-4 under autotrophic and mixotrophic conditions

#### Microscopic observation of mutant Z-4 with Nile red staining

**Fig. 1:**
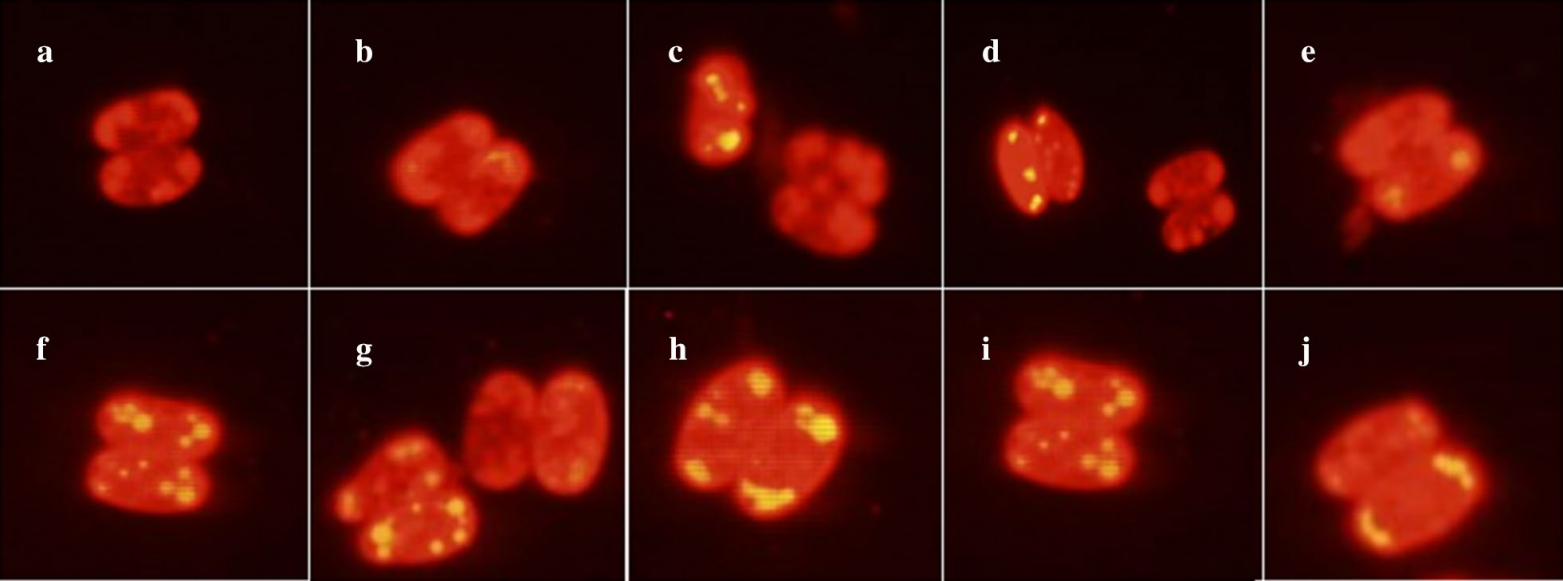
Fluorescence photographs of microalgal mutant *Scenedesmus* sp. Z-4 staining by Nile red at different light/dark cycles under autotrophic and mixotrophic conditions. **a**–**e** autotrophic cultivation; **f** heterotrophic cultivation; **g**–**j** mixotrophic cultivation. **a**, **f** light/dark cycle of 0 h/24 h, **b**, **g**: light/dark cycle of 6 h/18 h, **c**, **h** light/dark cycle of 12 h/12 h, **d**, **i** light/dark cycle of 18 h/6 h, **e**, **j** light/dark cycle of 24 h/0 h

The fluorescence intensity and amount of lipid droplets in cells from microalgal mutant *Scenedesmus* sp. Z-4 using Nile red staining at different light/dark cycles of 0 h/24 h, 8 h/16 h, 12 h/12 h, 16 h/8 h, and 24 h/0 h under autotrophic and mixotrophic conditions are shown in Fig. 1a–j. Under autotrophic cultivation, the fluorescence intensity and amount of lipid droplets in microalgal cells increased with the increase of light duration from 0 to 12 h, and decreased with further increase of light duration to 24 h. Hardly any yellow fluorescence was observed at the light/dark cycle of 0 h/24 h, suggesting that light was an essential ecological factor for lipid accumulation of microalgal mutant Z-4 (Fig. 1a). However, excess exposure to light did not seem beneficial to lipid synthesis (Fig. 1d, e). Compared to autotrophic cultivation, intracellular fluorescence intensity in all tests under mixotrophic cultivation was obviously stronger (Fig. 1f–j). The largest amount of lipid droplets was found when the light/dark cycle was 12 h/12 h (Fig. 1h), and this phenomenon was similar to that under autotrophic cultivation. Carbon dioxide (CO_2_) could be converted into glucose in the dark reaction process of photosynthesis, which produces the precursor for lipid production, Acetyl-CoA, via glycolysis under the two cultivation modes. Therefore, the dark environment was an indispensable factor for lipid synthesis. However, photoinhibition and photosaturation due to long duration of light irradiation will lead to a decline in photosynthetic efficiency, which is not favorable to the accumulation of lipid as storage substance. The above results verified that a certain time period of dark conditions was beneficial to lipid production of microalgal cells.

### Cell growth and lipid production of mutant Z-4

**Fig. 2:**
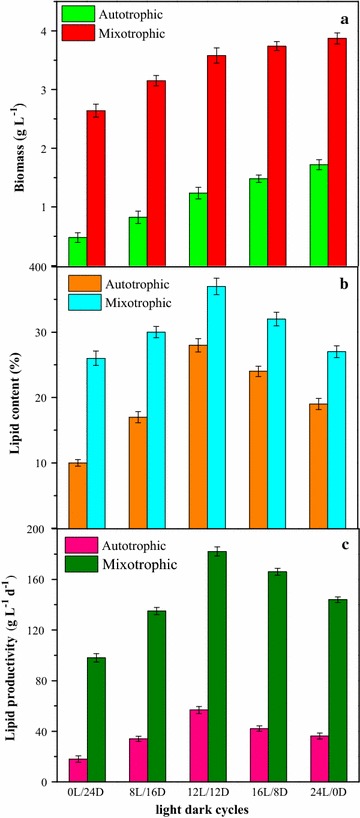
Biomass and lipid accumulation of microalgal mutant *Scenedesmus* sp. Z-4 at different light/dark cycles under autotrophic and mixotrophic conditions

The biomass and lipid productivity of mutant Z-4 at different light/dark cycles were determined under autotrophic and mixotrophic conditions (Fig. 2a). The results showed that, under autotrophic cultivation, biomass increased from 0.47 to 1.68 g L^−1^ as the light duration increased from 0 (0 h/24 h) to 24 h (24 h/0 h). This demonstrated that prolonging light time was beneficial to promote cell growth and biomass accumulation. Photosynthesis is the basis of survival and growth for autotrophically cultured cells, and it is activated by light energy. In the photoreaction stage, extension of the irradiation time could facilitate the production of more ATP and [H] for synthesizing organic substances in the dark reaction stage. Therefore, biomass could be accumulated evidently at longer light times. Faster growth rate and higher cell density of *Dunaliella salina* CCAP 19/30 could be obtained under continuous light (24 h/0 h), compared to that under other light/dark cycles [25]. The same trend in both studies occurred that longer light duration could enhance microalgal growth, even though *Scenedesmus* and *Chlorella* belong to different microalgal species. However, the biomass obtained in this study was higher than that in most reported studies under autotrophic conditions [26–28], and atmospheric CO_2_ was used in this work instead of concentrated CO_2_ with volume percentage of 5, 14.1, and 15%. This may be due to superior lipid production ability of mutant strain Z-4, which could effectively absorb and convert CO_2_ into lipid via cellular metabolism, even at low CO_2_ concentrations. By contrast, biomass was much higher under mixotrophic conditions, and gradually increased from 2.84 to 3.86 g L^−1^ with increasing irradiation time from 0 to 24 h. When light duration increased from 16 to 24 h, the enhancement of biomass almost stopped, indicating that superfluous light time (16–24 h) in each light/dark cycle did not further promote the biomass of mixotrophically cultured cells. This meant that light/dark cycles had a more remarkable influence on cell growth under autotrophic cultivation than mixotrophic cultivation. The reason could be attributed to different cellular metabolism pathways of the two cultivation modes. Light was the only energy source sustaining microalgal growth via photosynthesis under autotrophic conditions, but the growth of mixotrophic cells mainly depended on the supply of organic carbon sources, including glucose, acetate, glycerol. In addition, the higher cell densities under mixotrophic conditions could be due to the larger amount of available energy, mainly from biological processes of aerobic respiration coupled with catabolism of glucose and photosynthesis [29]. However, for mixotrophic cultures, biomass accumulation did not depend on light but relied on substrate concentration and microalgal species.

The continuous increase of light duration was not in favor of lipid accumulation under autotrophic or mixotrophic conditions (Fig. 2b, c). This corresponded to the above results from Nile red staining. When light irradiation time increased from 0 (0 h/24 h) to 12 h (12 h/12 h), lipid content obviously increased from 17.3 to 28.1% under autotrophic conditions, indicating that extension of light time was beneficial to lipid synthesis. Excessive light irradiation time inhibited the lipid production, and lipid content declined to 19.4% as the light irradiation time further increased to 24 h. Longer light irradiation time could cause the phenomenon of light saturation [30], in which superfluous light photons are not absorbed and converted by pigment molecules. In addition, the coupling of photoreactions and dark reactions does not match well due to the photoreaction acceleration. Therefore, the reduced photosynthetic efficiency was unfavorable to production of reserve substances such as lipids. However, lipid content was almost constant under five different light/dark cycles (as in our experimental set) for *Nannochloropsis gaditana* [31]. This difference may be due to different microalgal species and effects of light saturation. Moreover, the trend of lipid accumulation under mixotrophic conditions was in agreement with that under autotrophic condition. The maximum lipid content of 37.8% was obtained at the light/dark cycle of 12 h/12 h, indicating that excessive light time had a negative effect on lipid production of mutant Z-4.

The tendency of calculated lipid productivity at different light/dark cycles was similar to that observed lipid content (Fig. 2c). The maximum lipid productivities of 56.8 mg L^−1^ day^−1^ under autotrophic conditions and 182.6 mg L^−1^ day^−1^ under mixotrophic conditions were obtained under the light/dark cycle of 12 h/12 h. The values were obviously higher than those under other light/dark cycles of 0 h/24 h, 8 h/16 h, 16 h/8 h, and 24 h/0 h (18.2/34.6/42.1/36.2 mg L^−1^ day^−1^ for autotrophic cultivation, and 97.7/135.3/166.2/142.6 mg L^−1^ day^−1^ for mixotrophic cultivation, respectively). The biomass and lipid productivity of mutant Z-4 and some microalgal strains at different light/dark cycles in relevant studies are listed in Table 1, and the results indicated the great potential of mutant Z-4 in biodiesel production due to its superior lipid synthesis capability.

**Table 1:** Comparison of biomass and lipid productivity between *Scenedesmus* sp. Z-4 and some microalgal strains with different light/dark cycles in previous studies

Strain	Cultivation mode	Light/dark cycle	Biomass (g L^−1^)	Lipid productivity (mg L^−1^ day^−1^)	References
*Scenedesmus obliquus*	Autotrophic	24 h/0 h	0.992	Not given	[8]
*Chlorella sorokiniana*	Mixotrophic	12 h/12 h	1.55	31.62	[17]
*Chlorella* sp.	Autotrophic	16 h/8 h	0.63	12.6	[18]
*Nannochloropsis* sp.	Mixotrophic	16 h/8 h	1.10	42.31	
*Chlorella vulgaris*	Autotrophic	Not given	0.25	4	[19]
	Mixotrophic		1.70	54	
*Dunaliella salina*	Autotrophic	24 h/0 h	1.46	Not given	[25]
*Chlorella* sp.	Autotrophic	24 h/0 h	0.64	Not given	[26]
*Tetraselmis suecica*	Autotrophic	24 h/0 h	0.72	Not given	
*Scenedesmus* sp. Z-4	Autotrophic	12 h/12 h	1.68	56.8	This study
	Mixotrophic		3.86	182.6	

### Fatty acid composition of mutant Z-4

The composition of fatty acids for mutant Z-4 at different light/dark cycles under autotrophic cultivation was determined (Additional file 1: Table S1). The main fatty acids in all tests consisted of palmitic acid (C 16:0), stearic acid (C 18:0), oleic acid (C 18:1 w9c), and linoleic acid (C 18:2 w6,9c), making up more than 90% of total fatty acids. The results showed that with light duration increasing from 0 to 24 h, total saturated fatty acids gradually increased and total unsaturated fatty acids decreased. Especially, when light irradiation increased from 12 to 18 h, an obvious drop could be found in the amount of saturated fatty acids. This could be attributed to the influence of photooxidation caused by excessive light time leading to excessive dissolved oxygen accumulation. Therefore, the hydroxyl radicals produced in this process may promote the transformation of saturated fatty acids into unsaturated fatty acids. More saturated fatty acids (C 16:0 and C 18:0) and less unsaturated fatty acids (C 18:2 and C 18:3) have been confirmed to be suitable for biodiesel production [32]. Therefore, extension of light duration was not in favor of the quality of fatty acids.

The composition of fatty acids under mixotrophic condition was also affected by light/dark cycles, and an obvious decrease in content of saturated fatty acids was observed with light time increasing from 0 to 24 h (Additional file 1: Table S2). The composition was almost the same at different light/dark cycles of 0 h/24 h, 8 h/16 h, and 12 h/12 h, indicating that synthesis of different fatty acids by mixotrophic cells may mainly depend on utilization of organic carbon sources, and effects of light duration on the compositions were almost negligible. However, effects including photooxidation became eminent when the light irradiation time increased to a higher value.

Here, biomass, lipid content, and productivity and composition of fatty acids at different light/dark cycles have been summarized in Table 2, which is helpful for understanding the effects of light/dark cycle on autotrophically and mixotrophically cultured cells intuitively. A conclusion could be reached that light/dark cycles had a more remarkable effect on microalgal cells under autotrophic conditions. The appropriate light irradiation time could promote cell growth and lipid production; however, an excessive light period could inhibit lipid synthesis and change the composition of fatty acids. Therefore, considering the integrated results of biomass, lipid productivity, and composition of fatty acids, the optimal light/dark cycle for mutant Z-4 was determined to be 12 h/12 h.

**Table 2:** A summary of biomass, lipid content, and productivity and composition of fatty acids at different light/dark cycles under autotrophic and mixotrophic conditions

	Autotrophic condition					Mixotrophic condition				
	0 h/24 h	8 h/16 h	12 h/12 h	16 h/8 h	24 h/0 h	0 h/24 h	8 h/16 h	12 h/12 h	16 h/8 h	24 h/0 h
Biomass (g L^−1^)	0.48	0.82	1.23	1.48	1.72	2.74	3.15	3.58	3.74	3.87
Lipid content (%)	10.23	17.14	28.32	24.35	19.24	26.41	30.12	37.68	32.27	27.24
Lipid productivity (mg L^−1^ day^−1^)	18.42	34.65	56.82	42.13	36.26	98.13	135.62	182.64	166.31	144.67
Saturated fatty acids (%)	56.41	54.55	53.41	48.41	47.41	53.95	53.55	53.48	49.59	46.58
Unsaturated fatty acids (%)	43.59	45.45	46.59	51.59	52.59	46.05	46.45	46.52	50.59	53.52

### Characterization of cellular metabolism at different light/dark cycles under autotrophic and mixotrophic conditions

#### Chlorophyll content and photosynthetic efficiency

**Fig. 3:**
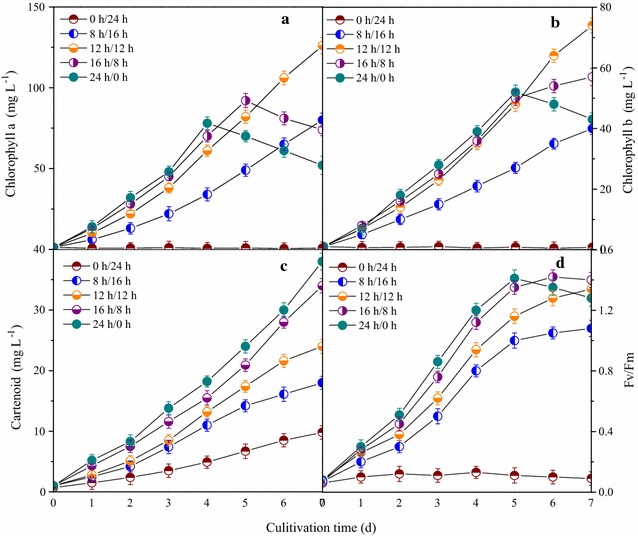
Effects of light/dark cycles on accumulation of pigment molecules and photosynthetic efficiency. **a** chlorophyll a; **b** chlorophyll b; **c** carotenoid; **d** photosynthetic efficiency

The effects of light/dark cycles on accumulation of pigment molecules including chlorophyll a, chlorophyll b, and carotenoid, which play important roles in the process of photosynthesis, were investigated under autotrophic conditions (Fig. 3a–c). The results showed that there was almost no production of chlorophyll a at the light/dark cycle of 0 h/24 h (Fig. 3a), indicating light was an indispensable factor for the production of chlorophyll a. The content of chlorophyll a continually increased to the maximum of 81.2 and 126.4 mg L^−1^ on the 7th day at the light/dark cycles of 8 h/16 h and 12 h/12 h, which could be verified by the phenomenon that the color of the microalgal cells of mutant Z-4 changed from yellow green to dark green in the process of cultivation (pictures not shown here). Higher synthetic rates of chlorophyll a could be observed at the light/dark cycles of 16 h/8 h and 24 h/0 h, which manifested that longer light time in each light/dark cycle (16–24 h) was favorable to rapid accumulation of chlorophyll a. However, an obvious decrease in content of chlorophyll a occurred at these two light/dark cycles, leading to the obtained maximum of 92.1 and 77.8 mg L^−1^ on the 5th and 4th day, respectively. This may be due to decomposition of chlorophyll a caused by higher input of light energy. On one hand, the molecule of chlorophyll a is unstable and easily broken under higher light intensity [33]. On the other hand, two key enzymes involved in biological synthesis of chlorophyll a, glutamyl-tRNA reductase (GluTR), and glutamate 1-semialdehyde aminotransferase (GSA-AT) are sensitive to light and their activity could be inhibited by excessive exposure to light [34].

The substrate for biosynthesis of chlorophyll b is derived from chlorophyll a; therefore, hardly any production of chlorophyll b could be detected at the light/dark cycle of 0 h/24 h in the cultivation process, because no chlorophyll a was converted into chlorophyll b (Fig. 3b). The total content of chlorophyll b gradually increased to peak values of 41.0 mg L^−1^ (0 h/24 h), 74.3 mg L^−1^ (12 h/12 h), and 56.2 mg L^−1^ (16 h/8 h) obtained on the 7th day. It is worth noting that the production of chlorophyll b at the light/dark cycle of 16 h/8 h started to slow down in the late cultivation period, and the content of chlorophyll b at the light/dark cycle of 24 h/0 h continually decreased from the 5th to 7th day. This may be due to dependence of chlorophyll b biosynthesis on chlorophyllide a oxygenase (CAO), which is highly expressed in low light [35].

Even though synthesis of carotenoid is not in need of light energy, the carotenoid content of microalgal mutant *Scenedesmus* sp. Z-4 at the light/dark cycle of 0 h/24 h was limited by poor biomass (Fig. 3c). The production of carotenoid in all tests exhibited a gradual increasing tendency, and the final carotenoid content was 9.8 mg L^−1^ (0 h/24 h), 18.2 mg L^−1^ (8 h/16 h), 24.1 mg L^−1^ (12 h/12 h), 34.3 mg L^−1^ (18 h/6 h), and 38.5 mg L^−1^ (24 h/0 h). An obvious improvement of carotenoid accumulation could be observed from the 5th to 7th day at light/dark cycles of 16 h/8 h and 24 h/0 h. On the one hand, the biomass produced under longer light time could provide abundant substrate for biosynthesis of carotenoid. More importantly, carotenoid, as an important antioxidant in microalgal cells, could be largely synthesized to alleviate the unfavorable effects of hydroxyl radical (·OH) when cells are exposed to longer light time. This may be a self-protective mechanism of mutant Z-4.

The maximum photochemical efficiency of PS II (*F*v/*F*m) for microalgal mutant Z-4 with different light/dark cycles under autotrophic conditions was determined (Fig. 3d). The value of *F*v/*F*m was almost zero in the process of cultivation when the light/dark cycle was 0/24 h, indicating that the photosynthetic system (PS) could not be motivated without light energy. *F*v/*F*m gradually increased to peak values on the 7th day at the light/dark cycles of 8 h/16 h and 12 h/12 h, and results in these two groups showed that prolonging irradiation time could promote the activity of PS. However, *F*v/*F*m no longer increased from the 5th to 7th day when the light/dark cycle of 18 h/6 h was used, which was mainly due to effects of light saturation. More importantly, photoinhibition took effect on the 5th to 7th day at the light/dark cycle of 24 h/0 h, leading to an obvious decline in photochemical efficiency.

Effects of light/dark cycles on accumulation of pigment molecules and photochemical efficiency were explored under mixotrophic conditions (Additional file 1: Figure S1). Compared to those under autotrophic conditions, the main differences are summarized in the following aspects. First, more chlorophyll a, chlorophyll b, and carotenoid could be produced under mixotrophic conditions. There is no doubt that addition of glucose was beneficial to microalgal anabolism, providing convenience for synthesis of cellular components. Second, effects of excessive light irradiation time on production of pigment molecules under mixotrophic conditions were not as strong as those under autotrophic conditions. These results may be attributed to resistance to photoinhibition due to relatively active metabolism under mixotrophic cultivation, and this needs to be investigated in future work. Last, the values of *F*v/*F*m were lower under mixotrophic conditions than those under autotrophic conditions. Growth of microalgal cells under mixotrophic conditions mainly relied on supply of organic carbon sources, thus the photosynthesis of mixotrophically cultured cells was not active compared to that of autotrophically cultured cells. Therefore, reduced photochemical efficiency of PS II could be observed, which is in accordance with a previously reported study [20].

### Substrate utilization under mixotrophic conditions

**Fig. 4:**
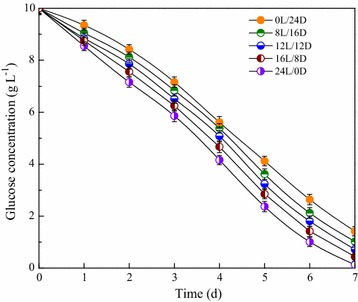
Time course profiles of glucose utilization for microalgal mutant Z-4 at different light/dark cycles under mixotrophic cultivation

The effects of light/dark cycles on glucose consumption of microalgal mutant Z-4 were explored (Fig. 4). With the increasing light duration in each light/dark cycle, the glucose utilization rate gradually increased. In the process of 7 days’ cultivation, the utilization rate of glucose at light/dark cycle of 0 h/24 h was approximately 87%; however, the final glucose concentration at the light/dark cycle of 24 h/0 h was almost zero. The results indicated that longer light irradiation time could promote glucose utilization by microalgal cells. Therefore, more biomass could be obtained under continuous light (24 h/0 h), as shown in Fig. 2a. To date, no studies about effects of light on substrate utilization of microalgal cells have been reported. The results in this work may be due to the influence of photosynthesis, in which the produced ATP and [H] were beneficial to the metabolic activity of mixotrophically cultured cells. With the acceleration of photoreaction due to prolonged light time, more ATP and [H] will participate in cellular anabolism, therefore strengthening the need for substrate consumption.

### Variation of cellular components

**Fig. 5:**
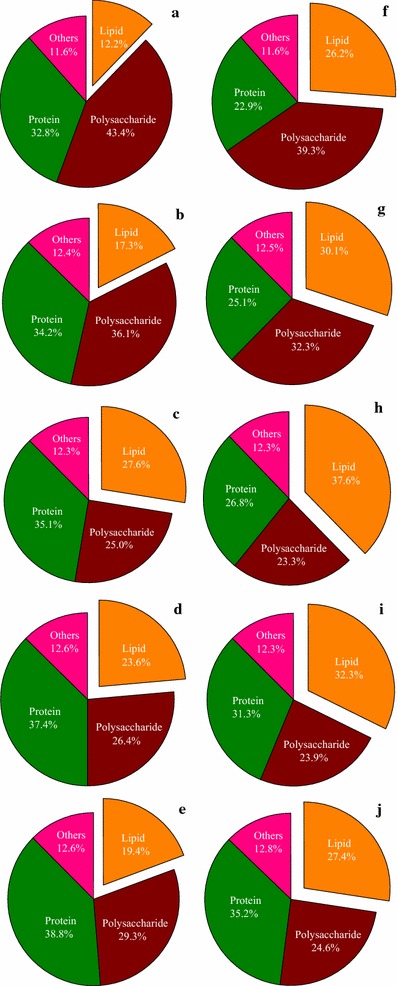
Percentage of main cellular components of microalgal mutant Z-4 at different light/dark cycles. **a**–**e** autotrophic cultivation; **f** heterotrophic cultivation; **g**–**j** mixotrophic cultivation. **a**, **f** light/dark cycle of 0 h/24 h, **b**, **g** light/dark cycle of 6 h/18 h, **c**, **h** light/dark cycle of 12 h/12 h, **d**, **i** light/dark cycle of 18 h/6 h, **e**, **j** light/dark cycle of 24 h/0 h)

To investigate the cellular metabolism of microalgal mutant Z-4 at different light/dark cycles, the percentage of main cellular components under autotrophic and mixotrophic conditions was determined (Fig. 5). Polysaccharide occupied a high proportion of 43% at the light/dark cycle of 0 h/24 h for autotrophically cultured mutant Z-4 (Fig. 5a). In another study, starch largely accumulated under adverse growing conditions to maintain cellular survival [36], which may explain the above results with no provision of light energy. As the light duration increased from 0 to 12 h, the percentage of polysaccharide decreased, leading to an increase in the contents of lipid and protein (Fig. 5b, c). This meant that when metabolism activities were motivated via photosynthesis by light, intracellular storage substance (such as starch) began to convert into other components. However, further increasing the light time to 16 and 24 h was unfavorable to lipid accumulation (Fig. 5d, e), as discussed above. It is worth noting that protein levels continually increased, which may be attributed to relatively active metabolism with the extension of light time. Therefore, more protein could be produced to implement cellular functions.

The contents of the main cellular components under mixotrophic condition at diverse light/dark cycles were also different (Fig. 5f–j), indicating that the light/dark cycle played an important role in cellular metabolism, even though light was not the primary energy for mixotrophic cells. Similarly, the percentage of lipid and protein gradually increased and the content of polysaccharide decreased with increasing light time from 0 to 12 h (Fig. 5f–h). This was because light energy could promote glucose consumption and catabolism (Fig. 4), which was beneficial to the synthesis of lipid and protein. However, with the further increase of irradiation time, the percentage of polysaccharide was almost constant, and a proportion of lipid was converted into protein (Fig. 5i, j). The results could be ascribed to the production of functional proteins due to excessive light irradiation, which may relieve unfavorable effects on cell growth.

### Cell growth, lipid production, and glucose utilization under the combined effects of temperature variation and light/dark cycle

#### Cell growth

**Fig. 6:**
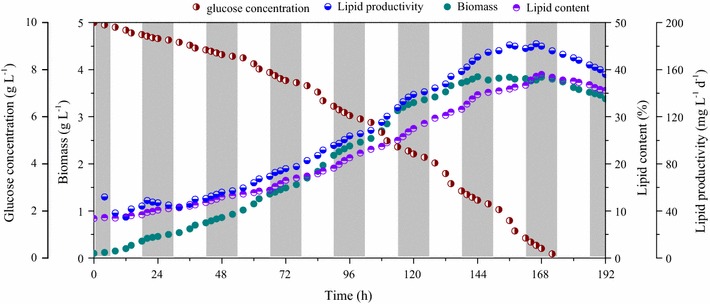
Cell growth, lipid production, and glucose consumption under the combination of temperature variation and light/dark cycle. The rectangular regions which are marked with white or gray represent light and dark conditions, respectively

The combined effects of temperature variation and light/dark cycles on cell growth of microalgal mutant *Scenedesmus* sp. Z-4 in a cultivation process of 8 days were investigated (Fig. 6). The results showed that the microalgal cells were at lag phase in the first 48 h, when the biomass concentration gradually increased at a low growth rate. Afterwards, mutant Z-4 entered into the logarithmic phase (48–144 h), and a considerable increase of biomass could be observed. On the 7th day, microalgal biomass almost reached a standstill, and it started to decrease in the following 24 h.

Furthermore, the microalgal cells exhibited more vigorous growth when exposed to light, compared to that under dark conditions. Especially, substantial enhancement in biomass concentration was shown in the daytime of the 3rd, 4th, and 5th day (54–66 h, 78–90 h, and 102–114 h), when biomass productivity was 0.76, 1.18, and 1.26 mg L^−1^ day^−1^, respectively. However, the corresponding biomass productivity values under dark condition during the same cultivations period were 0.34 mg L^−1^ day^−1^ (66–78 h), 0.60 mg L^−1^ day^−1^ (90–102 h), and 0.56 (114–126 h) mg L^−1^ day^−1^. On one hand, this could be attributed to abundant concentration of substrates (glucose, NaNO_3_, and K_2_HPO_4_, etc.) in the medium. On the other hand, the light in the daytime could further promote cellular metabolism, leading to a rapid rate of cell growth and large accumulation of cellular components. In particular, an obvious increase of biomass could be obtained during 56–62, 80–86, and 104–110 h (in light cycles), mainly due to relatively higher temperature. This meant that properly increasing the temperature under light conditions was beneficial to biomass production. However, the cell growth rate greatly decreased during the cultivation time of 46–54, 70–78, and 94–102 h (in dark cycles), which was probably the result of inactive metabolism under low temperature and dark conditions.

### Lipid accumulation

The accumulation of lipid content with the extension of cultivation time differed from that of biomass concentration (Fig. 6). The results showed that the lipid content maintained at a lower level (10–15%) in the first 72 h, because the produced biomass was not adequate to synthesize lipid. When the biomass concentration increased to approximately 2.0 g L^−1^, the lipid content began to increase and noticeable lipid accumulation could be observed during 90–102, 114–126, 138–150, and 162–168 h (in dark cycles), while the increase of lipid content under light conditions was not remarkable. This implied that lipid synthesis mainly occurred at late logarithmic phase and stationary phase under dark cycles. Considering the biomass production occurring under light conditions, an assumption could be made that a portion of the biomass produced during the day was utilized to synthesize lipid under dark conditions. Therefore, providing a certain time of dark conditions in the process of microalgal cultivation may be beneficial to lipid production. However, this concept may be species-dependent.

In addition, the effects of temperature variation on lipid accumulation were also shown. Lipid content greatly increased at the beginning 4 h for each dark cycle (especially 90–94, 114–118, 138–142, 162–166 h), when the temperature gradually decreased from 15 to 5 °C. The results may be ascribed to the special cellular metabolism of microalgal mutant Z-4. When the microalgal cells went into a dark cycle, biomass accumulation started to slow down and lipid synthesis through biomass conversion became active with the decrease of temperature. Therefore, lipid content obviously increased in the first hours of the dark cycles. However, as the temperature further decreased to 0 °C, cellular metabolism was severely inhibited, and the conversion of biomass to lipid was not remarkable. When the temperature gradually increased, this conversion became active again.

The accumulation of lipid productivity with the prolonging of cultivation time is also illustrated in Fig. 6. The lipid productivity was an integrated result of biomass and lipid production. The results indicated that no obvious enhancement in lipid productivity could be observed in the first 72 h, because the biomass concentration and lipid content remained at a relatively low level. After 72 h of cultivation time, due to evident biomass accumulation under light cycles and lipid production under dark cycles, lipid productivity increased continually, reaching a maximum of 182.6 mg L^−1^ day^−1^ at 166 h of cultivation. Afterwards, lipid productivity gradually decreased due to an obvious decrease of biomass concentration, which demonstrated that excessively prolonging the cultivation time was not beneficial to improvement of lipid productivity.

### Glucose utilization

To explore substrate utilization in the process of biomass accumulation, the time course profile of glucose concentration under the combined effect of temperature and light/dark cycles is depicted in Fig. 6. The results showed that glucose consumption was relatively slow in the first 48 h. This was due to, on one hand, inactive cellular metabolism at the stationary phase. On the other hand, the ratio of glucose concentration to cellular density was higher, so excessive glucose had an inhibitory effect on cell growth. This explanation is rational because the enhancement of biomass concentration was rather limited, even though the glucose concentration in the medium was abundant. When the microalgal cells entered into logarithmic phase, the glucose consumption rate sped up to satisfy cellular survival and breeding. Furthermore, a similar phenomenon could be found in that massive glucose was utilized in the daytime of the 4th, 5th, and 6th day (78–90, 102–114, 126–138 h), when the glucose concentration decreased from 7.42 to 6.45, 5.85 to 4.72, and 4.16 to 2.85 g L^−1^, respectively. This implied that biomass accumulation coincided well with substrate consumption, and these two processes mainly occurred in the beginning of light cycles, when the gradually increasing temperature and provided illumination were in favor of cell growth and substrate uptake.

Therefore, an important conclusion could be reached that biomass accumulation of strain Z-4 was mainly accomplished under light conditions via glucose utilization. The suitable temperature and illumination promoted the increase of biomass concentration. When microalgal cells turned into dark conditions, lipid started to synthesize by the conversion of biomass. These results could provide a significant reference for outdoors lipid-derived biodiesel production by mutant strain Z-4.

## Conclusions

The effects of light/dark cycles (0 h/24 h, 8 h/16 h, 12 h/12 h, 16 h/8 h, 24 h/0 h) on cell growth and lipid accumulation of the oleaginous microalgae *Scenedesmus* sp. mutant Z-4 were investigated in batch culture. The results indicated that when the optimal light/dark cycle of 12 h/12 h was applied in the experiments, the maximum lipid productivity of 56.8 and 182.4 mg L^−1^ day^−1^ could be obtained under autotrophic and heterotrophic conditions, respectively. Moreover, both insufficient and excessive light time were unfavorable to lipid accumulation. Cellular metabolism under autotrophic cultivation was remarkably affected by light/dark cycles due to severe dependence on light. Furthermore, maximum biomass of 3.74 g L^−1^ and lipid productivity of 172.4 mg L^−1^ day^−1^ could be obtained under the combined effects of temperature variation and light/dark cycle. These results could provide important clues for cell growth and lipid production of strain Z-4 under outdoor conditions.

## Methods

### Microalgal species and culture conditions

The previously isolated microalgal strain *Scenedesmus* sp. Z-4 was applied in the experiments. This strain was routinely cultured under batch culture in 250 mL Erlenmeyer flasks in a shaker at constant temperature (30 °C), which contained 150 mL BG-11 medium (its ingredients could be found everywhere). In general, 10% of microalgal inoculum (15 mL) at late logarithmic phase was transferred into fresh medium, which was adjusted to pH of 7.0 and autoclaved at 121 °C for 15 min, to start a new round of cultivation. Besides, microalgal cells were cultivated at 3000 lx by white fluorescent light with the light/dark cycle of 12 h/12 h.

### Experimental design

In this study, autotrophic and mixotrophic conditions were established to study the effects of light/dark cycles on cell growth and lipid production of mutant Z-4 under these two conditions. Under autotrophic cultivation, Erlenmeyer flasks containing 150 mL medium were sealed with a piece of parafilm, which allowed gas exchange, and no additional carbon source was provided. Under mixotrophic conditions, microalgal cultivation was supplied with 10 g L^−1^ glucose in BG-11 medium and light energy simultaneously.

Furthermore, five different light/dark cycles of 0 h/24 h, 8 h/16 h, 12 h/12 h, 18 h/6 h, and 24 h/0 h were applied in this experiment, which could be controlled by an artificial climate chamber (HPG-280H, China). To explore outdoors cell growth and lipid production of the strain under conditions of temperature variation and light/dark cycle, the chosen highest and lowest temperatures were 25 and 0 °C, respectively. The daytime started at 6:00 and ended at 18:00 every day to form a light/dark cycle of 12 h/12 h. The concrete temperature settings and sampling times are shown in Additional file 1: Figure S1. The biomass concentration and lipid content of samples, and glucose concentration in the medium, were determined every several hours in the cultivation time of 192 h.

### Analytical methods

Biomass was collected by centrifugation, washed with normal saline, and weighed after drying to constant weight for several hours. Lipid was extracted by chloroform/methanol (2:1, v/v) and determined according to the procedures in the literature [37]. Biomass and lipid productivity could be calculated by using the following equation [4]:

1 $${\text{Biomass productivity}}\;\left( {{\text{mg}}\;{\text{L}}^{ - 1} \;{\text{day}}^{ - 1} } \right) = {{\left( {b_{2} - b} \right)} \mathord{\left/ {\vphantom {{\left( {b_{2} - b} \right)} {\left( {t_{2} - t} \right)}}} \right. \kern-0pt} {\left( {t_{2} - t} \right)}}$$

2 $${\text{Lipid productivity}}\;\left( {{\text{mg}}\;{\text{L}}^{ - 1} \;{\text{day}}^{ - 1} } \right) = {{{\text{biomass }}\; \times \; {\text{lipid content}}} \mathord{\left/ {\vphantom {{{\text{biomass }}\; \times \; {\text{lipid content}}} t}} \right. \kern-0pt} t}.$$

Here, *b*_*2*_ and *b*_*1*_ are the biomass at the cultivation times of *t*_*2*_ and *t*_*1*_. *t* is the cultivation time (day), equal to 7 days for this strain.

The analysis of fatty acid composition was performed by gas chromatography [38]. The solvent phase was combined, dried, and weighed. The glucose concentration in the medium was determined by the oxidase method [7]. The contents of polysaccharide and protein in microalgal cells were measured by previous methods [39, 40]. Chlorophyll a, chlorophyll b, and carotenoid were extracted with 80% acetone, and their contents were determined by spectrophotometry according to the following equations [41]:

3 $${\text{Chl-a}}\;\left( {{{\upmu {\text{g}}} \mathord{\left/ {\vphantom {{\upmu {\text{g}}} {\text{mL}}}} \right. \kern-0pt} {\text{mL}}}} \right) = 16.72\;{\text{OD}}_{{665.2\;{\text{nm}}}} {-}9.16\;{\text{OD}}_{{652.4\;{\text{nm}}}}$$

4 $${\text{Chl-b}}\;\left( {{{\upmu {\text{g}}} \mathord{\left/ {\vphantom {{\mu {\text{g}}} {\text{mL}}}} \right. \kern-0pt} {\text{mL}}}} \right) = 34.09\;{\text{OD}}_{{652.4\;{\text{nm}}}} {-}15.28\;{\text{OD}}_{{665.2\;{\text{nm}}}}$$

5 $${\text{Carotenoid}}\;\left( {{{\upmu {\text{g}}} \mathord{\left/ {\vphantom {{\mu {\text{g}}} {\text{mL}}}} \right. \kern-0pt} {\text{mL}}}} \right) = {{\left( {1000\;{\text{OD}}_{{ 4 7 0\;{\text{nm}}}} {-}1.63\;{\text{Chl-a}}{-}104.9\;{\text{Chl - b}}} \right)} \mathord{\left/ {\vphantom {{\left( {1000\;{\text{OD}}_{{ 4 7 0\;{\text{nm}}}} {-}1.63\;{\text{Chl-a}}{-}104.9\;{\text{Chl - b}}} \right)} {221}}} \right. \kern-0pt} {221}}.$$

All experiments were conducted in triplicate, and the standard deviation (SD) was presented. Cells stained with Nile Red (dissolved in acetone solution, 0.1 mg mL^−1^) were observed with a fluorescence microscope (BX51/TF, Olympus Co., Japan), and the excitation wavelength of 530 nm and emission wavelength of 575 nm were applied. The maximum photochemical quantum yield of PS II reaction centers was reflected by *F*v/*F*m (the variable/maximum fluorescence ratio) [42].

## Additional file

**Additional file 1.** **Figure S1.** Effects of light/dark cycles on accumulation of pigment molecules and photosynthetic efficiency under mixotrophic condition. **Figure S2.** Temperature variation applied in this study. **Table S1.** The compositions of fatty acids (mass percentage) of microalgal mutant Z-4 at different light–dark cycles under autotrophic condition. **Table S2.** The compositions of fatty acids (mass percentage) of microalgal mutant Z-4 at different light–dark cycles under mixotrophic condition.

## Abbreviations

CO_2_: carbon dioxide
TCA: tricarboxylic acid cycle
acetyl-CoA: acetyl coenzyme A
GluTR: glutamyl-tRNA reductase
GSA-AT: glutamate 1-semialdehyde aminotransferase
CAO: chlorophyllide a oxygenase
OH: hydroxyl radical
PS: photosynthetic system
*F*v: variable fluorescence
*F*m: maximum fluorescence
OD: optical density
SD: standard deviation
ATP: adenosine triphosphate
