# Supplementary material for: Cell growth and lipid accumulation of a microalgal mutant Scenedesmus sp. Z-4 by combining light/dark cycle with temperature variation
Source: Biotechnol Biofuels. 2017 Nov 9;10:260. doi: 10.1186/s13068-017-0948-0 (PMC5679341; doi:10.1186/s13068-017-0948-0)
Supplement: Supplementary file 1 — Additional file 1. Figure S1. Effects of light/dark cycles on accumulation of pigment molecules and photosynthetic efficiency under mixotrophic condition. Figure S2. Temperature variation applied in this study. Table S1. The compositions of fatty acids (mass percentage) of microalgal mutant Z-4 at different light–dark cycles under autotrophic condition. Table S2. The compositions of fatty acids (mass percentage) of microalgal mutant Z-4 at different light–dark cycles under mixotrophic condition. [file 13068_2017_948_MOESM1_ESM.doc]

**Additional file 1**

**Cell growth and lipid accumulation of a microalgal mutant *Scenedesmus* sp. Z-4 by combining light/dark cycle with temperature variation**

Chao Ma, Yan-Bo Zhang, Shih-Hsin Ho, De-Feng Xing, Guo-Jun Xie, Nan-Qi Ren, Bing-Feng Liu^*^

*State Key Laboratory of Urban Water Resource and Environment, School of Municipal and Environmental Engineering, Harbin Institute of Technology, P.O. Box 2614, 73 Huanghe Road, Harbin 150090, China*

*Email:lbf@hit.edu.cn*

**Fig. S1 Effects of light/dark cycles on accumulation of pigment molecules and photosynthetic efficiency under mixotrophic condition**

**Fig. S2 Temperature variation applied in this study. The rectangular regions which are marked with white or grey represent light and dark conditions, respectively. The purple points are for sampling and determination of biomass and lipid content.**

**Table S1 The compositions of fatty acids (mass percentage) of microalgal mutant Z-4 at different light-dark cycles under autotrophic condition**

| Compositions of fatty acids | 0/24 h | 8 h/16 h | 12 h/12 h | 16 h/8 h | 24 h/0 |
| --- | --- | --- | --- | --- | --- |
| C 15:0 2OH | 0.67 ± 0.12 | - | 0.58 ± 0.13 | 0.60 ± 0.13 | 0.54 ± 0.12 |
| C 15:0 3OH | 0.20 ± 0.07 | - | 0.22 ± 0.07 | 0.22 ± 0.07 | 0.20 ± 0.07 |
| C 16:0 | 38.98 ± 2.03 | 37.51 ± 3.28 | 36.98 ± 3.56 | 35.98 ± 3.56 | 34.98 ± 2.03 |
| C17:1 w8c | - | 0.22 ± 0.10 | - | - | - |
| C18:1 w7c | 0.43 ± 0.09 | 0.25 ± 0.09 | 0.43 ± 0.11 | 0.43 ± 0.11 | 0.43 ± 0.09 |
| C18:1 w9c | 25.92 ± 1.64 | 27.92 ± 2.34 | 28.43 ± 2.62 | 28.43 ± 2.62 | 25.92 ± 1.64 |
| 18:2 w6,9c | 18.22 ± 1.35 | 13.50 ± 1.13 | 14.24 ± 1.64 | 14.24 ± 1.64 | 18.22 ± 1.35 |
| 18:3 w6c (6,9,12) | 1.86 ± 0.59 | 1.90 ± 0.31 | 2.86 ± 0.28 | 3.16 ± 0.28 | 3.86 ± 0.59 |
| C 20:0 | 0.30 ± 0.11 | 0.28 ± 0.07 | 0.29 ± 0.10 | 0.29 ± 0.10 | 0.30 ± 0.11 |
| C 20:1 w9c | 0.17 ± 0.04 | 0.18 ± 0.06 | 0.17 ± 0.08 | 0.17 ± 0.08 | 0.17 ± 0.04 |
| Saturated fatty acids | 56.41 ± 2.33 | 54.55 ± 3.35 | 53.41 ± 3.86 | 48.41 ± 3.86 | 47.41 ± 2.33 |
| Unsaturated fatty acids | 43.59 ± 3.71 | 45.46 ± 4.03 | 46.59 ± 4.73 | 51.59 ± 4.73 | 53.59 ± 3.71 |

**Table S2 The compositions of fatty acids (mass percentage) of microalgal mutant Z-4 at different light-dark cycles under mixotrophic condition**

| Compositions of fatty acids | 0/24 h | 8 h/16 h | 12 h/12 h | 16 h/8 h | 24 h/0 |
| --- | --- | --- | --- | --- | --- |
| C 15:0 2OH | 0.68 ± 0.12 | - | 0.61 ± 0.13 | 0.58 ± 0.13 | 0.52 ± 0.12 |
| C 15:0 3OH | 0.20 ± 0.07 | - | 0.18 ± 0.07 | 0.22 ± 0.07 | - |
| C 16:0 | 38.98 ± 2.03 | 38.51 ± 3.28 | 37.98 ± 3.56 | 36.98 ± 3.56 | 35.98 ± 2.03 |
| C17:1 w8c | - | 0.22 ± 0.10 | - | 0.20 ± 0.04 | - |
| C18:1 w7c | 0.43 ± 0.09 | 0.25 ± 0.09 | 0.43 ± 0.11 | 0.43 ± 0.11 | 0.43 ± 0.09 |
| C18:1 w9c | 25.92 ± 1.64 | 27.92 ± 2.34 | 28.43 ± 2.62 | 28.43 ± 2.62 | 25.92 ± 1.64 |
| 18:2 w6,9c | 18.22 ± 1.35 | 13.50 ± 1.13 | 14.24 ± 1.64 | 14.24 ± 1.64 | 18.22 ± 1.35 |
| 18:3 w6c (6,9,12) | 3.86 ± 0.59 | 1.90 ± 0.31 | 1.86 ± 0.28 | 1.86 ± 0.28 | 3.86 ± 0.59 |
| C 20:0 | 0.30 ± 0.11 | 0.28 ± 0.07 | 0.29 ± 0.10 | 0.29 ± 0.10 | 0.30 ± 0.11 |
| C 20:1 w9c | 0.17 ± 0.04 | 0.18 ± 0.06 | 0.17 ± 0.08 | 0.17 ± 0.08 | 0.17 ± 0.04 |
| Saturated fatty acids | 53.95 ± 2.33 | 53.55 ± 3.35 | 53.48 ± 3.86 | 49.59 ± 3.86 | 46.48 ± 2.33 |
| Unsaturated fatty acids | 46.05 ± 3.71 | 46.45 ± 4.03 | 46.52 ± 4.73 | 50.41 ± 4.73 | 53.52 ± 3.71 |
